# Dataset on outdoor behavior-system and spatial-pattern in the third place in cold area-based on the perspective of new energy structure

**DOI:** 10.1016/j.dib.2016.12.040

**Published:** 2016-12-29

**Authors:** Kai Ren, Yuan Wang, Tingxi Liu, Guanli Wang

**Affiliations:** a Water conservancy and Civil Engineering College, Inner Mongolia Agriculture University, Zhaowuda Road No.306, Hohhot, Inner Mongolia, China; b Inner Mongolia Power CO. LTD. Hohhot, Inner Mongolia, China

**Keywords:** The third place, Cold area, Behavior system, Spatial pattern, Symbiosis theory

## Abstract

The data presented in this paper are related to the research article entitled “Exploration of Outdoor Behavior System and Spatial Pattern in the Third Place in Cold Area- based on the perspective of new energy structure” (Ren, 2016) [1]. The dataset was from a field sub-time extended investigation to residents of Power Home Community in Inner Mongolia of China that belongs to cold region of ID area according to Chinese design code for buildings. This filed data provided descriptive statistics about environment-behavior symbiosis system, environment loading, behavior system, spatial demanding and spatial pattern for all kinds of residents (Older, younger, children). The field data set is made publicly available to enable critical or extended analyzes.

**Specifications table**

**Table:** 

Subject area	Environment, Behavior
More specific subject area	Residents’ outdoor behaviors in cold climate.
Type of data	Tables, Figures, Text file
How data was acquired	Behavior-system were observed by field sub-time extended investigation to residents;
	Spatial pattern were observed by questionnaire, measuring and mathematical statistics.
Data format	Raw, Analyzed
Experimental factors	Publicly available data sources except cold climate.
Experimental features	Relationship between behavior and environment was assumed symbiotic, and measurement method of environment was given.
Data source location	Hohhot, Inner Mongolia, China, 40 °29׳28.01" N, 111 °47׳07.69" E
Data accessibility	The data are available within this article.

**Value of data**

1. The data presents the factors of environment-behavior relationship in outdoors, such as environmental load, seasonal differences, historical images, spatial prototypes, behavior types, occurrence space, occurrence time, etc.
2. The data presents the measurement method of symbiotic level between environment and behaviors.
3. Other researchers may find the data useful for different types of analysis in areas such as urban planning.
4. The data are publicly available, but are dispersed within several different sources.

## 1 Data

**Fig. 1:**
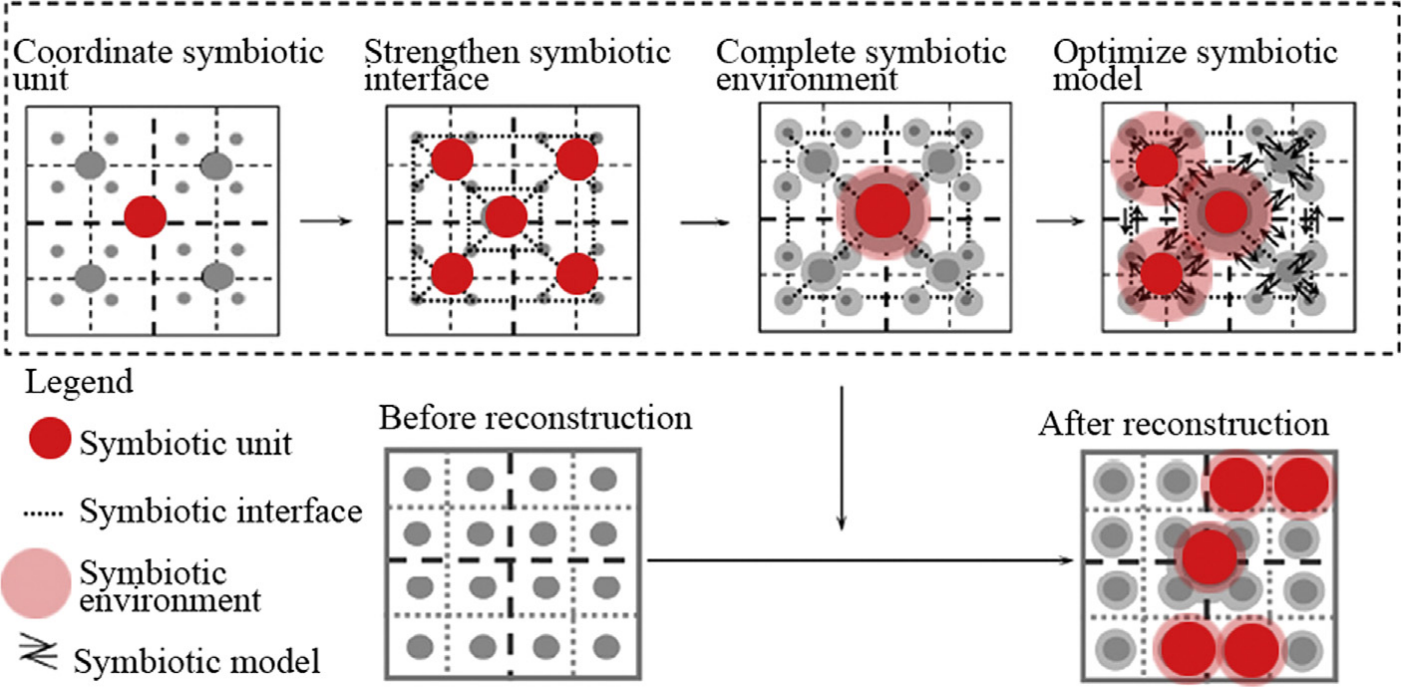
Spatial pattern set up by space syntax tools.

The dataset of this paper provides information on environment-behavior symbiosis system, environment loading, behavior system and spatial demanding for all kinds of residents (Older, younger, children) [2], [3]. Table 1, Table 2, Table 3, Table 4, Table 5 showed the relationship between environment and behaviors, and Fig. 1 showed the spatial pattern.

## 2 Experimental design, tools and methods

The dataset was from a field sub-time extended investigation to residents of Power Home Community in Inner Mongolia of China that belongs to cold region of ID area according to Chinese design code for buildings [1]. Spatial form of the third places was done by Depth-map X software (space syntax method), and was completed according to line segment model of season circle [4], [5].

The data of environment-behavior symbiosis system was constructed as follows:

$$S=\sum_{i}^{n}\mathrm{Ti\cdot Ei\cdot Bi}(S=\mathrm{symbiosis},T=\mathrm{time},\;E=\mathrm{environment},\;B=\mathrm{behavior})$$

All the data were converted into definitions (High, medium, low) for the purposes of the analysis.

## Figures and Tables

**Table 1:** Components of symbiotic system for different groups.

**Symbiotic system**	**The aged**	**The young**	**Children**
**Symbiosis matrix**	Same behaviors: sitting, with grandson, walking the dog, shopping, etc.	Same behaviors: fitness, social connection, relaxation, shopping, etc.	Same behaviors: playing, sporting, etc.
**Symbiosis unit**	Age community (over 65 years old)	Age community (35–65 years old)	Age community (below 12 years old)
**Symbiosis interface**	Infrastructure, ancillary facilities, community facilities. (optioned by individuals)	Infrastructure, ancillary facilities, community facilities. (optioned by individuals)	Infrastructure, ancillary facilities, community facilities. (optioned by individuals)
**Symbiosis environment**	Space environment, rule environment. (depends on selective tendencies of crowd)	Space environment, rule environment. (depends on selective tendencies of crowd)	Space environment, rule environment. (depends on selective tendencies of crowd)

**Table 2:** Main constituent elements of outdoor environment and load proportion.

**Elements description**	**Green**	**Street**	**Fitness**	**Strolling**	**Sitting**	**Dog**	**Coffee**	**Playing**	**Peddler**
**Changing/repeating**	(C90%, R10%)	(C92%, R8%)	(C30%, R70%)	(C89%, R11%)	(C80%, R20%)	(C95%, R5%)	(C19%, R81%)	(C90%, R10%)	(C91%, R9%)
**Fresh/accustomed**	(F35%, A65%)	(F95%, A5%)	(F29%, A71%)	(F95%, A5%)	(F96%, A6%)	(F95%, A5%)	(F20%, A80%)	(F40%, A60%)	(F91%, A9%)
**Homogeneous/heterogeneous**	(I95%, H5%)	(I30%, H70%)	(I93%, H7%)	(I10%, H90%)	(I87%, H13%)	(I8%, H92%)	(I92%, H8%)	(I95%, H5%)	(I94%, H6%)
**Concentrated/loose**	(C68%, L32%)	(C59%, L41%)	(C45%, L55%)	(C37%, L63%)	(C30%, L70%)	(C28%, L72%)	(C40%, L60%)	(C12%, L88%)	(C75%, L25%)
**Large-scale/small-scale**	(L76%, S24%)	(L21%, S79%)	(L19%, S81%)	(L87%, S13%)	(L8%, S92%)	(L78%, S22%)	(L10%, S90%)	(L9%, S91%)	(L12%, S88%)
**Symmetrical/asymmetrical**	(S21%, A79%)	(S7%, A93%)	(S9%, A91%)	(S10%, A90%)	(S15%, A85%)	(S3%, A97%)	(S8%, A92%)	(S25%, A75%)	(S13%, A87%)
**Dynamic/stationary**	(D38%, S62%)	(D45%, S55%)	(D94%, S6%)	(D89%, S11%)	(D88%, S12%)	(D92%, S8%)	(D39%, S61%)	(D89%, S11%)	(D79%, S21%)
**Radom/modal**	(R20%, M80%)	(R22%, M78%)	(R13%, M87%)	(R90%, M10%)	(R80%, M20%)	(R77%, M23%)	(R19%, M81%)	(R69%, M31%)	(R88%, M12%)
**Nearby/farther**	(N65%, F35%)	(N70%, F30%)	(N69%, F31%)	(N39%, F61%)	(N92%, F8%)	(N24%, F76%)	(N80%, F20%)	(N36%, F64%)	(N65%, F35%)
**Walking/car**	(W95%, C5%)	(W90%, C10%)	(W87%, C13%)	(W96%, C4%)	(W90%, C10%)	(W90%, C10%)	(W79%, C21%)	(W90%, C10%)	(W88%, C12%)

**Table 3:** Survey and statistical analysis way of environment-behavior bidirectional relationship.

**Time line 1(12 valid time period per day)**	**Occurrence period 8:00–20:00(each hour is a period)**	**Occurrence period 8:00–20:00(each hour is a period)**	**Occurrence period 8:00–20:00(each hour is a period)**	**Occurrence period 8:00–20:00(each hour is a period)**
**Typical Communities surrounding constitutes**	Space of the third place S1	Space of the third place S2	Space of the third place S3	Space of the third place Sn
**The Aged**	Type A1	Type A2	Type A3	Type A4
**The Young**	Type Y1	TypeY2	TypeY3	Type Y4
**Children**	Type C1	Type C2	Type C3	C4
**Space Line(outdoor space category of the third place)**	Necessity space	Spontaneity space	Chain space	Passive space
**Time line 2(season)**	Occurring interval(SP, SU, AU, WI.)	Occurring interval(SP, SU, AU, WI.)	Occurring interval(SP, SU, AU.)	Occurring interval(SP, SU, AU.)

**Table 4:** Outdoor selective behavior types and space types of different groups (The old/The young/Children) in cold region and their season changing.

Crowd type	Old/Young/Children (O/Y/C)	(O/Y/C)	(O/Y/C)	(O/Y/C)	(O/Y/C)	(O/Y/C)
Distribution period	**8:00~10:00**	**10:00~12:00**	**12:00~14:00**	**14:00~16:00**	**16:00~18:00**	**18:00~20:00**
Behavior types						
A spontaneous activity	**Higher incidence (15%)**	**Highest incidence (30%)**	**Lower incidence (10%)**	**Highest incidence (30%)**	**Lower incidence (10%)**	**Lowest incidence (5%)**
B necessary activities	**Highest Incidence (30%)**	**Lower incidence (10%)**	**Higher incidence (15%)**	**Higher incidence (15%)**	**Higher incidence (15%)**	**Higher incidence (15%)**
C chain activities	**Lowest incidence (5%)**	**Lowest incidence (5%)**	**Lower incidence (10%)**	**Highest incidence (30%)**	**Highest incidence (30%)**	**Higher incidence (20%)**
Space Types						
Walking space	**O/Y prefer**	**O/C prefer**	**Y prefer**	**Y prefer**	**O/Y/C uniform**	**O/Y prefer**
Stay space	O/Y prefer	O/Y prefer	Y prefer	O/Y prefer	O/C prefer	O/Y prefer
Chatting space	**O/Y prefer**	**O/C prefer**	**O/Y prefer**	**O/C prefer**	**O/Y prefer**	**O/Y/C uniform**
Flexible space	O/Y/C uniform	O/Y/C uniform	O/Y/C uniform	O/Y/C uniform	O/Y/C uniform	O/Y/C uniform
Season changing	**SP and SU**	**SP and AU**	**WI**	**WI**	**SP and AU**	**SU**

**Table 5:** Definitions (high, medium, low) analyzed by space syntax and multiple functions.

Analytical method	Assay value	Definition
Space syntax	High	1. The selectivity assay values of global and microscopic scales are both high. 2. One of the selectivity assay value of global and microscopic scales is high, and another is medium.
	Medium	1. The selectivity assay values of global and microscopic scales are both medium. 2. One of the selectivity assay value of global and microscopic scales is high, and another is low.
	Low	1. The selectivity assay values of global and microscopic scales are both low. 2. One of the selectivity assay value of global and microscopic scales is medium, and another is low.
Mixed function indicator	High	1. Three kinds of functions mixing.
	Medium	1. Two kinds of functions mixing.
	Low	1. Single function.
