# Supplementary material for: Dataset on outdoor behavior-system and spatial-pattern in the third place in cold area-based on the perspective of new energy structure
Source: Data Brief. 2016 Dec 29;10:593–7. doi: 10.1016/j.dib.2016.12.040 (PMC5226851; doi:10.1016/j.dib.2016.12.040)
Supplement: Supplementary file 1 — Supplementary material [file mmc1.docx]

**Conflict of Interest Form**

We wish to draw the attention of the Editor to the following facts which may be considered as potential conflicts of interest and to significant financial contributions to this work. We confirm that the manuscript has been read and approved by all named authors and that there are no other persons who satisfied the criteria for authorship but are not listed. We further confirm that the order of authors listed in the manuscript has been approved by both of us. We confirm that we have given due consideration to the protection of intellectual property associated with this work and that there are no impediments to publication, including the timing of publication, with respect to intellectual property. We understand that the Corresponding Author is the sole contact for the Editorial process (including Editorial Manager and direct communications with the office). She is responsible for communicating with the other authors about progress, submissions of revisions and final approval of proofs. We confirm that we have provided a current, correct email address which is accessible by the Corresponding Author and which has been configured to accept email from [dib@elsevier.com](mailto:dib@elsevier.com).

Signed by all authors as follows:

Kai Ren: [renkai921724girl@sina.cn](mailto:renkai921724girl@sina.cn)

Yuan Wang: [wangyuan126126@163.com](mailto:wangyuan126126@163.com)
